# Measuring patient and family perceptions of team processes and outcomes in healthcare teams: questionnaire development and psychometric evaluation

**DOI:** 10.1186/s12913-018-3808-0

**Published:** 2019-01-06

**Authors:** Kelley Kilpatrick, Éric Tchouaket, Lysane Paquette, Claudel Guillemette, Mira Jabbour, François Desmeules, Véronique Landry, Nicolas Fernandez

**Affiliations:** 1 Susan E. French Chair in Nursing Research and Innovative Practice, Ingram School of Nursing, McGill University, Montréal, Canada; 2 Centre intégré universitaire de santé et de services sociaux de l’Est-de-l’Île-de-Montréal-Hôpital Maisonneuve-Rosemont (CIUSSS-EMTL), Montréal, Canada; 3 Université du Québec en Outaouais, Saint-Jérôme, Canada; 4 Faculty of Nursing, Université de Montréal, Montréal, Canada; 5 School of Rehabilitation, Faculty of Medicine, Université de Montréal, Montréal, Canada; 6 Faculty of Medicine, Université de Montréal, Montréal, Canada

**Keywords:** Inter-professional, Nurse practitioner, Patient-reported experience measure, Patient-reported outcome, Perceptions of team effectiveness, Process, Questionnaire development, Reliability, Team functioning, Validity

## Abstract

**Background:**

There is a lack of validated instruments examining dimensions of team functioning from the perspective of patients and families consistent with a conceptual framework. The study aimed to develop and assess the psychometric properties of the Patient-Perceptions of Team Effectiveness (PTE) questionnaire.

**Methods:**

A cross-sectional survey was undertaken in three studies. Data were collected from May–October 2016 for Study 1, April 2018-ongoing for Study 2, and October 2016 to June 2017 for Study 3. Online and paper versions of the self-administered questionnaire were available in English and in French. The initial questionnaire included 41 items. Study 1 included 320 respondents. Reliability was assessed using Cronbach alpha. Face validity (*n* = 250) was assessed using a structured questionnaire. Content validity was examined using subject matter experts and Spearman’s item-total correlations. Construct validity was examined using known group comparisons (i.e., clinical specialty, education, length of follow-up, reason of consultation). Content analysis was used for open-ended questions.

**Results:**

The questionnaire took 10 to 15 min to complete. Positive assessments were noted for instructions, formatting, font size and logical ordering of questions. In Study 1, reliability indices for the PTE-Overall, Team Processes and Outcomes subscales ranged from 0.72 to 0.84. Item-total correlations ranged from 0.551 to 0.794 (*p* <  0.001). Differences were noted between clinical specialties, education, length of follow-up, reason of consultation, low and high functioning teams. No differences were noted between English and French language respondents. Psychometric properties were re-assessed in Study 2 and 3 after unclear questions were reworked. Reliability indices for the subscales ranged from 0.76 to 0.94 and differences remained significant between low and high functioning teams.

**Conclusion:**

The final 43-item instrument is easy to administer to patients and families. The studies provide evidence of validity to support the propositions in the conceptual framework. The patient-level measures can be aggregated to the team, organizational or system level. The information can be used to assess healthcare team functioning in acute and primary care and determine the role patients and families are playing in teams. Further testing is needed with patients and families who are hospitalized or receiving care from teams in rural areas.

## Background

Internationally, there is increased interest to implement patient-reported experience measures (PREMs) to report on what happens during a healthcare visit and improve the effectiveness of care processes [1]. Patient-reported outcome measures (PROMs) examine the impact of health conditions from the patient’s perspective [2]. PREMs and PROMs provide complementary information and are being used to examine healthcare system performance and care quality [3–6]. Researchers with the Belgian Health Care Knowledge Centre [7] completed a review of systematic reviews (*n* = 11) of PREMs and PROMs published after 2010, and found that much of the literature was focused in oncology and on PROM measures.

Wiering et al. [8] identified 193 PROMs in specialized and primary care following a scoping review of the PROM literature. Studies using generic PROMs (e.g., EQ-5D) have been of limited use because of their lack of responsiveness and ability to measure change [9]. There has been limited consensus on appropriate PROMs to use in primary care to capture the overall outcome of a visit, as opposed to disease-specific outcomes, especially for patients with several chronic conditions [10, 11]. Similarly, Desomer et al. [7] concluded in their review of reviews that research has focused largely on the impact of PROMs on the patient clinician relationship but all other domains such as primary care and chronic conditions are understudied. They identify a lack of standardized PREM measures, and measures that are at the individual and hospital level but not at the level of the healthcare team as important limitations. Patient judgements about how their healthcare team functions are team-level PREM measures [12].

Casimiro and colleagues [13] highlighted that care providers within the team need to remain flexible and shift teamwork patterns from uni- to interprofessional to facilitate patient-engaged care. This underscores the need for patients and families to understand how different providers contribute to their care. Team processes including communication, involvement in decision-making, cohesion, care coordination, and problem-solving help to explain how something is done or how events unfold over time within a specific context [14, 15]. These processes are dynamic and adaptive actions or steps undertaken by patients or providers to achieve a specific goal [16]. However, we have a very limited understanding of processes in interprofessional teams because it is “*like looking into a black box”* ([17] p.20, [18], p. S11).

Teams are defined as two or more individuals who work interdependently toward a common and valued goal [19]. Delivery models that promote interprofessional care are believed to be the most effective way to provide healthcare services [20]. Studies have demonstrated that improvements in teamwork can improve patient safety and efficiency in different settings including the operating theatre [21], palliative care [22, 23], and cardiopulmonary arrest simulations [24, 25]. Interprofessional healthcare teams were introduced in hospitals over a century ago [26]. However, interprofessional teams are relatively new in primary care [27], and their evaluation is a global imperative [28].

There is some empirical evidence to support the link between processes and outcomes in healthcare teams [29, 30]. In acute care, Tremblay et al. [31] found that high levels of inter-professional teamwork influenced PREMs in cancer care and Sidani and Doran [32] found some evidence to link processes to outcomes for nurse practitioners (NPs) in acute care. In primary care, Beaulieu et al. [33] examined organizational characteristics associated with high quality primary care and found that provider perceptions of work group innovations were moderately associated with outcomes. Poulton and West [34] demonstrated that processes influenced 23% of change in patient outcomes after controlling for different primary care structures. More recently, Strasser et al. [35] examined five measures of team functioning in rehabilitation teams and determined that only three out of 15 process measures of team functioning were sufficiently sensitive to identify changes in patient outcomes. These authors [35] concluded that team functioning measures were in their infancy but they showed promise and warranted further investigation. El Ansari et al. [36] developed a team effectiveness scale to measure team functioning in adult mental health teams in the community. The methodology used allowed them to generate context-specific measures of effectiveness for teams in the United Kingdom.

The impact of poorly functioning teams is staggering. In the United States (US), a review of over 23,000 malpractice claims across a range of practices over four years found that miscommunication was a factor in more than half the claims, costing $1.7 billion US and leading to the loss of 2000 lives [37]. There is a growing interest in understanding how processes in teams influence patient outcomes and patient experience of care [31, 38, 39]. Castro et al. [40] completed a concept analysis to distinguish between patient empowerment, patient participation and patient-centeredness in hospital care. These authors argued that patient centeredness occurs at the micro level of patient and provider interactions. Evidence is emerging to document the relationship between team structures, processes, perceived care quality and patient experience in cancer care [39].

To date, researchers [41] have focussed primarily on patient views of individual-level interactions with providers (e.g., communication, care coordination) to better understand patient experience and patient engagement [42]. Research is now beginning to shift to the team-level and focus on the patient as a partner in the healthcare team [43]. We know little about micro-level processes in healthcare teams or how they support the development of the role of patients and families as team members [40, 44]. Jeffcott and Mackenzie ([45], p., 190) argued that team performance is difficult to measure but there is a recognition of the “*need to advance team measurement*.” Valentine, Nembhart, and Edmonson [46] completed a systematic review of instruments to measure teamwork. These authors highlighted that existing questionnaires did not measure key dimensions (e.g., communication, coordination) of team functioning that were consistent with a conceptual framework.

There is a large body of research examining teamwork, interprofessional education, patient safety and communication in different healthcare settings (e.g., AHRQ, 2017; Brandt & Schmidtz, 2017; IOM, 2015) [47–49]. High quality evidence including systematic reviews have determined that care delivered by interprofessional teams (e.g., nurses, physicians, physiotherapists, nutritionists, etc.) improved patient outcomes (e.g., Andreatta & Marzano, 2012; Epstein, 2014: Rutherford, 2017) [50–52]. The quality of patient experiences of care has been identified as a “critical indicator of healthcare performance” ([53], p.17). However, Beaird et al. [54] and Pomey, Ghadiri, Karazivan, Fernandez, and Clavel [43] argued that more work was needed to include patients in the team.

Patients and families are able to identify and assess provider behaviours related to teamwork [55]. As an exemplar, patients and families under the care of interprofessional teams that included NPs believed their team was more effective after the implementation of an NP regardless if they received services in acute or primary care [44]. NPs have additional training and competencies compared with nurses allowing them to prescribe medications and treatments, order and interpret tests, and perform clinical procedures [56]. Patients and families described how teams with NPs used specific processes (e.g., communication, care coordination) to effectively provide care. Although patients and families identified similar processes across healthcare settings, the relative importance of each process changed if patients were cared for by teams in acute or primary care [44].

There is limited research examining how interprofessional teams provide care [14, 35, 57]. Research is needed to determine the influence of different structures, micro-level processes in primary and acute care teams on outcomes using validated measures [30, 58].To help fill this gap in knowledge, valid and reliable instruments are needed to assess team processes from the perspective of patients and families in acute and primary care.

### Conceptual framework

The study was supported by the conceptual framework developed specifically to explain how roles are enacted and interprofessional teams function [59]. Three central processes are at the heart of the framework including role enactment, boundary work and perceptions of team effectiveness. Role enactment in teams focuses on what providers do in their role on a day-to-day basis and on how care is provided. Role enactment is influenced by role clarity. Boundary work is a process of shifting or negotiating new professional boundary lines between members of the team when a new role is introduced or roles change in the healthcare team. Boundary work activities evolve over time. The development of trust among team members is a key factor that influences boundary work activities and changes in professional roles [60]. Perceptions of team effectiveness include the beliefs that the team can perform across a range of activities and team members want to continue working together. In separate studies that examined patient, family and provider perceptions of team effectiveness, participants identified six processes in effective teams [41, 61]. They included improved communication, involvement in decision-making, cohesion, care coordination, problem-solving, and a focus on patient and family needs. Structures from the patient to the healthcare system level are represented in the framework using concentric circles that surround the central processes. Structures and processes influence outcomes such as quality, safety, costs and team outcomes. Relationships are symbolized by bi-directional arrows and dotted lines [59]. Within the framework and in the present study, the term interprofessional was used to examine interactions that involved members of more than one professional group (e.g., the nursing group and another professional group other than nursing). The conceptual framework were used to structure the questionnaire, and link structures, team processes and outcomes.

## Methods

### Aims

The study aimed to 1) develop and cross-validate English and French language versions of the Patient-Perceptions of Team Effectiveness (Patient-PTE) Questionnaire to assess team processes and perceived outcomes in healthcare teams from the perspectives of patients and families; and 2) assess the psychometric properties (i.e., reliability, face validity, content validity, and construct validity) of the Patient-PTE. We used the approach proposed by Dillman et al. [62] and Peter et al. [63] to develop and administer the questionnaires. This approach includes, among others, patient interviews, item generation, expert interviews, pilot-testing of the draft to identify unclear items and rephrasing, translation, and the pilot study to examine internal consistency and item correlations. The paper reports on the preliminary validation study (Study 1) and subsequent studies (Study 2 and Study 3 described below) that assessed the psychometric properties of the revised questionnaire.

### Instrument development

To address Objective 1, the questionnaire was developed using empirical evidence from studies examing team processes that included a descriptive qualitative study of patients and families (*n* = 49) under the care of acute and primary care teams with NPs [44], multiple-case studies of processes in interprofessional teams [60, 64, 65] and a literature review of processes in healthcare teams [14]. In the qualitative descriptive study [44], patients and families used examples of how care was provided by healthcare teams with and without NPs to identify or explain the similarities and differences with how care was provided by these teams. Patients and families had similar views of what constituted good or poor team functioning. The interviews allowed us to determine that one questionnaire was needed to examine team functioning from the patient and family perspective.

### Item generation

Items were generated using data from semi-structured interviews with patients and families that were transcribed verbatim and analyzed using content analysis [44]. We searched for descriptions of micro-level processes and actions identified by patients and families including how team members worked together to solve problems and if patients and families were involved in decisions about their care. We mapped the statements to processes and outcomes in teams to ensure that key processes in the framework were represented [66]. Draft items were developed from the statements and reviewed by members of the research team including a patient (NF) to ensure item clarity and legibility. In the qualitative descriptive study [44], we found that patients and families could name the professionals they included in their healthcare team to estimate the size of the team but several patients and families had difficulties distinguishing between professional groups, particularly between different groups of nurses (e.g., licensed practical nurses, registered nurses and nurse practitioners) in their healthcare team. Thus, one item was included to identify team size and another item ascertained if teams included a specific provider (e.g., nurse practitioners in the validation study). No item was developed to identify the professional groups in the team because of patient and family difficulties to distinguish between provider groups.

### Instrument

#### Patient-Perceptions of Team Effectiveness Questionnaire

The preliminary Patient-PTE questionnaire included 41 items. Section I describing the characteristics of the healthcare team included 6 items (e.g., type of healthcare setting, location, team size). The PTE-Overall subscale included 14 items divided into Team Processes (e.g., communication, decision-making) and the factors (e.g., trust, role clarity) known to influence these processes. The Outcomes subscale included five items (e.g., timely care, patient knowledge about medication). Responses in PTE-Overall and Outcomes used a 1–7 Likert scale. Responses ranged from *strongly disagree, disagree, disagree somewhat, neutral/no opinion, agree somewhat, agree,* and s*trongly agree*. The items were summed for each dimension (e.g., communication, coordination) and a mean value was estimated to obtain a score for the dimension (See Table 1). The scores ranged from 1 to 7. Higher scores in PTE-Overall, Team Processes and Outcomes indicated improved perceptions of team processes and outcomes respectively. Section IV describing demographic characteristics of patients and families included 15 items (e.g., perceived health, age, gender, education, marital status). Almost all response options in Section IV were categorical with the exception of length of follow-up which was a numeric value. One open-ended question was included to gather any additional comments from respondents. Single-item measures were used to assess boundary work (i.e., trust), belief about team effectiveness (BE), care coordination, and problem-solving [67]. Two-item measures were used to examine decision-making, communication, cohesion, patient and family focus, and role clarity. To reduce response burden, single-item measures may be more appropriate in vulnerable populations [67, 68].

**Table 1:** Preliminary Items Related to Healthcare Team Processes and Outcomes in Study 1

Item	
Role clarity	There is a lot of overlap between the roles of members of my healthcare team
	I am happy with the way work is divided in the team
Boundary work	Team members do not trust other members of the healthcare team
Perceptions of team effectiveness	My healthcare team is effective to provide healthcare
	Team members provide relevant information to help me make decisions about my healthcare
	The plan of care and care objectives are clearly outlined in my chart
	The notes in my health record are up to date
	The flow of information between team members and patients and families is constrained
	Team members work together to solve patient care issues
	Team members work in isolation (in silos)
	My ideas, information or observations are valued by members of my healthcare team
	Differences of opinion among team members are respected
	My role in the team is not important
	Working with families to solve patient care issues is not part of the team’s mandate
Outcome of Care	Patient care is delivered in a timely manner
	Potential or actual complications are dealt with quickly by the team
	I return home with as many unanswered questions about my medication
	After I have given my permission, all relevant information is available in my health record if I need to consult another healthcare provider or if I change units when I am hospitalized
	I return home with all my questions answered about my care

### Translation

Strategies to enhance the cross-cultural adaptation of the questionnaire included translation and back-translation by a professional translator and a review of the questionnaire by bilingual researchers [69]. We adopted the method proposed by Gillemin et al. [69]. Interview participants responded in English and French. We harmonized the statements into English. The initial questionnaire was translated from English to French and back-translated from French to English by back-translators who were unaware of the intent of the survey. The final translation was verified by the research team where four members are bilingual and have experience translating surveys. This allowed us to identify any discrepancies in translation [70].

Following the review of draft items by the research team, English and French language versions of the questionnaire were pre-tested with patients and families (*n* = 6) to identify unclear wording. As proposed by Kilpatrick et al. [71], the same participants completed the questionnaire after two weeks to determine if their understanding of the questions changed over time. One revision to improve clarity was made to one item following pre-testing (i.e., “Team members work in silos” was changed to “Team members work in isolation (in silos)”). No differences in participants’ responses and understanding of the questions were noted when participants completed the questionnaire after two weeks.

### Design

A cross-sectional survey were undertaken (Study 1). A self-administered questionnaire was identified as a cost-effective method to gather information from large groups of participants [72].

### Recruitment

In Study 1, a convenience sample was used. We aimed to recruit 300 patients and families to assess instrument performance and identify factors that influenced perceptions of team effectiveness. This sample size was sufficiently large to detect medium (0.30) correlations and a 95% confidence interval half-width of 0.1, a statistical power of 80% and a 5% alpha level [73, 74].

As proposed by Knafl, Leeman, Havill, Crandell, and Sandelowski [75], a broad definition of family was adopted to include persons who provide instrumental and relationship support deemed to be significant by the patient. Patients and families followed by teams in acute or primary care were eligible to participate in the study. Interviews with patients and families in primary care, nephrology, cardiology, and the neonatal intensive care unit indicated that they valued the same processes and perceived outcomes regardless of their health concern [44]. Nine to 10 participants in each group allowed us to attain theoretical and data saturation [44]. Acute care was defined as in-hospital or specialized ambulatory care to address specific health conditions [76]. Primary care was defined as the point of entry to the healthcare system and included comprehensive healthcare services for common health concerns [77]. Participants were recruited face-to-face in waiting rooms [78]. For this reason, it was not possible to estimate a response rate. It is worth noting that almost no participants declined to participate in the study. Those who declined or did not complete the questionnaire 1) were called in for their health appointment and did not have enough time to complete the questionnaire; or 2) they felt unwell or had received pre-medication prior to receiving their chemotherapy treatment. Questionnaires were administered to patients and families attending ambulatory acute care or outpatient care in a large integrated health system that included three hospitals, eight local community care centres, long-term care institutions, and day centres.

### Data collection

Following Research Ethics Committee approval (Study 1: 2016–712, 15,065; Study 2: MP-12-2017-841, CIUSSS-EMTL-203, Study 3: 2017–337), data were collected from May to October 2016 for Study 1. Online and paper copies of all study documents were available in English and in French to enhance response rates [79]. For Study 1, recruitment occurred in oncology, maternal and perinatal care, pediatrics, and primary care. Posters were placed in view in the waiting rooms of these clinics to advise potential participants that data collection was underway. An information sheet and consent form, a feedback form and questionnaire were included in each handout. Participants were advised that they were under no obligation to participate in the study and could withdraw at any time. To address Objective 2, cognitive interviewing, a feedback form, and statistical analyses were used to assess the psychometric properties of the questionnaire in Study 1.

### Cognitive interviewing

Cognitive interviewing with scripted probes were used to explore the meaning of participant responses [80]. Participants who completed the pre-test and the main survey were asked to describe their understanding of survey items (e.g., What did it mean to you when you responded…). Research assistants noted their responses to determine if their understanding matched the intent of the question [62]. As proposed by Kelley, Clark, Brown, and Sitzia [81], participants were asked to review instructions, questionnaire layout, procedures to complete French and English language questionnaires, and item and response options for clarity.

### Feedback form

As proposed by Pomey et al. [43], patients and families were viewed as subject matter experts. All participants in Study 1 were asked to complete a feedback form immediately following the completion of the questionnaire. Participants were asked how long it took to complete the questionnaire to identify a risk of response burden. The feedback form was used by Bryant-Lukosius et al. [82] and Kilpatrick et al. [71] to assess provider questionnaires. Participants were asked if 1) the time to complete the questionnaire was appropriate; 2) the instructions were clear; 3) relevant questions NOT included in the Patient-PTE questionnaire needed to be added; 4) there were any irrelevant, unimportant or redundant questions that could be removed without jeopardizing the completeness of the study results; 5 and 6) format and font size were easy to read; and 7) questions were ordered logically. The eighth item of the feedback form was an open-ended question and asked if patients and families had suggestions to improve the Patient-PTE questionnaire. Responses to the eighth question of the feedback form informed the assessment of content validity.

### Data analysis

Data were analyzed using IBM Statistical Package for the Social Sciences, version 25 [83]. Items were positively and negatively worded. The negatively-worded items (*n* = 7) were reverse-coded prior to the analyses. Descriptive statistics were generated (e.g., mean, standard deviation (SD), range). An average score was estimated to determine an overall Patient-PTE score (PTE-Overall). Scores by subscale and dimension were calculated. Criteria to assess the intrument’s performance included Cronbach alpha (α), item-total correlation of each item, and less than 5% missing data. The Shapiro-Wilk test was performed to test for normality [84]. Responses did not follow a normal distribution (*p* values < 0.001). Thus, Kruskal-Wallis one-way analysis of variance was used for non-parametric testing of variables with more than two categories and Mann-Whitney U was used to assess variables with two categories. The Mann-Whitney U post hoc test with a Bonferroni correction was applied where α was divided by the number of groups under consideration (e.g., α/3 = 0.017 for variables with three response categories) to correct for multiple comparisons and determine where the differences were between the groups. Spearman’s item-total correlations [rho coefficient] were used to assess the relationships for continuous data [85]. Content analysis was used for the open-ended questions and responses to cognitive interviews.

The numeric responses for length of follow-up were recategorized into less than 12 months, 12–59 months, and 60 months or more to identify differences in team processes over time. Education was recategorized as having completed or not completed high school education to ascertain if completion rates for the questionnaire were lower for respondents with less education. The responses to the item examining beliefs about team effectiveness (BE) from 1 to 5 were recategorized as low functioning teams and responses 6 and 7 for this item were recategorized as high-functionong teams. As proposed by Norman et al. (2003) [86] differences of half a standard deviation of the belief about team effectiveness score were considered to be a minimally significant difference in team functioning. After taking into account that data were not normally distributed and the small sample size in the low functioning group in the Study 2, we compared the median scores for Boundary Work, Team Processes, Role Clarity, and Outcomes between the groups using a Mann-Whitney U test.

### Instrument performance

*Reliability.* Internal consistency of the PTE-Overall, Team Processes and Outcomes subscales was assessed using Cronbach alpha (∝). Values ranging from 0.7 to 0.9 were considered acceptable to excellent [87]. For Study 1, the within-group variance was compared to the between-group variance to determine the consistency of responses among respondents within the same group [88]. As proposed by Verran, Gerber, and Milton [89], variability within the known groups should be less than the variability across the groups to aggregate data from the individual to the team or organizational level.

*Face validity* was assessed using the feedback form that examined the Patient-PTE questionnaire, formatting and instructions. Frequencies and percentages were generated.

*Content validity* was assessed in two steps. In the first step, prior to the questionnaire roll-out, different expert groups including patients/families (*n* = 6), researchers (*n* = 7), NP students (*n* = 25) and graduate students (*n* = 4) in other professional roles (e.g., physiotherapy, management) examined the questionnaire. Two questions were added to ask if respondents 1) had any one of 16 health conditions; and 2) the extent to which specific chronic conditions (i.e., diabetes, heart and respiratory problems) influenced their day-to-day activities. The second step occurred in Study 1 where all respondents were viewed as subject matter experts and asked to complete the open-ended question in the feedback form.

*Construct validity* was assessed using the known-group technique where we compared the scores between specific groups [90, 91]. Based on the views of patients and families who participated in the qualitative descriptive study of teams with NPs [44], we hypothesized a priori that no differences in scores would be found between 1) men and women; 2) patients and families; 3) English and French language respondents; 4) respondents in urban and rural locations; 5) marital status; and 6) employment status. Differences were expected according to 1) clinical speciality; 2) team size (small (less than 5 members), medium (5–10 members), large (more than 10 members); 3) respondents’ education; 4) length of follow-up; 5) reason of consultation (i.e., routine/annual examination, pregnancy follow-up, follow-up for a chronic health problem, new health problem, urgent health problem, several chronic health problems); and 6) teams with and without NPs. Factor analysis was not used to determine construct validity because the measures had fewer than three items per scale [92]. Responsiveness was defined as the instrument’s ability to detect a meaningful change [93]. Although there is no agreed-upon method to assess responsiveness, we hypothesized that the questionnaire could distinguish between low and high-functioning teams.

In the following section, results from Study 1 including means, correlations, Cronbach α and *p* values statistically significant at the 5% level or α/n_groups_ for post-hoc analysis are reported by measure and known group, followed by the results of Study 2 and 3.

## Results

Overall, 355 participants responded in Study 1. The questionnaire took 10 to 15 min to complete. We excluded 20 questionnaires that included only socio-demographic data and 15 questionnaires with more than 20% missing data for PTE-Overall and Outcomes. Thus, 320 questionnaires were included in the analyses. Almost all questionnaires were completed on paper; online questionnaires were completed by 5% of respondents (*n* = 17).

Socio-demographic characteristics of participants are presented in Table 2. Sixty seven percent (*n* = 209) of respondents were female, and their average age was 50.7 years ±15.9[mean ± SD]. Seventy nine percent (*n* = 248) of respondents were patients, and 39% (*n* = 121) of respondents perceived their health was good to very good (25%, *n* = 78). Most were born in Canada (79%, *n* = 242) and living in an urban area (97%, *n* = 309). Seventy three percent (*n* = 214) of respondents spoke only French and 82% (*n* = 262) of respondents completed the questionnaire in French. Seventy one percent (*n* = 222) of respondents were married or living with a partner. Eighty five percent (*n* = 266) had completed high school education or above. Thirty five percent (*n* = 109) of respondents worked full time.

**Table 2:** Characteristics of Respondents and Their Healthcare Team in Study 1

Characteristic	Variable		n	%
Respondent	Patient		248	79
	Family		65	21
	Language	English	58	18
		French	262	82
	Gender	Male	101	33
		Female	209	67
	Born in Canada	Yes	242	79
		No	66	21
	Marital status	Married/living with a partner	222	71
		Separated	10	3
		Divorced	16	5
		Widowed	12	4
		Never married	51	16
	Education	Did not complete high school	45	15
		Completed high school	62	20
		Some university education or completed a community or technical college	93	30
		Completed a bachelor’s degree (e.g., BA, BSc, BSN)	67	21
		Completed a graduate or professional degree (e.g., MD, PhD)	44	14
Healthcare Team	Length of follow up	< 12 months	150	51
		12–59 months	93	32
		≥60 months	51	17
	Location	Urban	309	97
		Non-urban	9	3
	Team size	Small	105	34
		Medium	108	35
		Large	93	30
	Nurse practitioner	Yes	32	10
		No/ Do not know	286	90

Respondents were followed by healthcare teams in oncology (64%, *n* = 205), maternal-perinatal care (17%, *n* = 55), primary care (9%, *n* = 28), pediatrics (5%, *n* = 17); and other specialties (5%, *n* = 14) including medicine, surgery, emergency-critical care, cardiovascular, rehabilitation, hospice/palliative care, community health, home care, psychiatry, mental health, and long-term care. The number of respondents in the primary care-pediatrics, maternal-perinatal care, and oncology groups were sufficiently large to allow for known-group comparisons.

Respondents consulted the healthcare team for a variety of reasons including new health problem, chronic illness care, and pregnancy follow-up. About half of respondents had been followed by their healthcare team for less than a year (51%, *n* = 150, 30 ± 53 months; range 1 to 420 months). The length of follow up by specialty was provided in Table 3. Most (90%, *n* = 286) were followed by healthcare teams that did not include an NP and about one third of participants viewed their team as a small, medium or large size team.

**Table 3:** Length of Follow-Up (Months) by Specialty in Study 1

Specialty Group	n^a^	Mean	Standard Deviation	Min-Max
Primary Care- Pediatrics	42	70.9	84.8	1–420
Maternal-Perinatal Care	51	14.1	30.3	1–156
Oncology	186	25.6	41.5	1–288
Total	279	30.3	51.8	1–420

^a^Fewer respondents due to missing data for specialty or length of follow-up

Reliability was assessed by estimating Cronbach α values for PTE-Overall, Team Processes and Outcomes. Cronbach α values for PTE-Overall and Team Processes equalled 0.84, and Outcomes equalled 0.72 (See Table 4). Across the groups, the Mean Square (MS) values for the within groups variability were much smaller than the MS values between groups for all the measures indicating that individual level data could be aggregated at the team or organizational level (See Table 5).

**Table 4:** Scores for Boundary Work, Perceptions of Team Effectiveness, Role Clarity, and Outcomes in Study 1

							Spearman’s item-total correlations with ‘Team Processes’	
Subscale Factor	Dimension	Number of items	Mean	Standard deviation	Min-Max	Cronbach alpha	rho coefficient	*p* value
Boundary Work	Trust	1	5.6	1.5	1–7			
Team Processes		11	5.4	0.9	1.6–7	0.84		
	Belief about team effectiveness	1	6.1	1.2	1–7		0.635	< 0.001
	Decision-making	2	5.5	1.3	1–7		0.754	< 0.001
	Communication	2	5.6	1.3	1–7		0.715	< 0.001
	Coordination	1	4.7	1.9	1–7		0.631	< 0.001
	Cohesion	2	5.6	1.2	1–7		0.794	< 0.001
	Problem-solving	1	5	1.3	1–7		0.551	< 0.001
	Patient focus	2	5.2	1.4	1–7		0.641	< 0.001
Role Clarity		2	5	1.3	1–7			
PTE-Overall		14	5.4	0.9	1.5–7	0.84		
Outcomes		5	5.4	1.1	1.6–7	0.72

**Table 5:** Between and Within-Group Variability by Specialty in Study 1

Subscale Factor	Variability	Sum of Squares	Degrees of freedom (df)	Mean Square
Boundary Work Trust	Between-groups	37.182	2	18.591
	Within-groups	605.595	298	2.032
	Total	642.777	300	
Team Processes	Between-groups	14.379	2	7.189
	Within-groups	242.760	302	0.804
	Total	257.139	304	
Role Clarity	Between-groups	15.148	2	7.574
	Within-groups	494.114	302	1.636
	Total	509.262	304	
PTE-Overall	Between-groups	15.736	2	7.868
	Within-groups	219.255	302	0.726
	Total	234.991	304	
Outcomes	Between-groups	14.720	2	7.360
	Within-groups	308.337	302	1.021
	Total	323.057	304	

Face validity was assessed using the responses (*n* = 250) in the feedback form (See Table 6). All the responses for the feedback form were completed using paper copies. Most (85%) believed that key questions to understand team functioning were included in the questionnaire and no question was unimportant. Positive assessments were noted for the time needed to complete the questionnaire, instructions, formatting, font size and logical ordering of questions.

**Table 6:** Responses Related to Face Validity in Study 1

		n	%
The time it took to complete the questionnaire was	Appropriate	242	96.7
	Too long	7	2.8
	Too short	1	0.4
Overall, the instructions contained in the consent were:	Clear	218	89
	Not clear	26	11
Are there any questions NOT included in this questionnaire that you feel would be important to include to better understand how teams function	No	204	85
	Yes	35	15
Are there questions you feel are unimportant, irrelevant, or redundant and could be eliminated from the questionnaire without jeopardizing completeness of the study results?	No	202	85
	Yes	36	15
The format of the questionnaire was	Easy to Read	227	95
	Difficult to read	12	5
The font size made the questionnaire	Easy to Read	231	97
	Difficult to read	6	3
The questions were ordered in a logical manner that was easy to follow	Agree	236	99
	Disagree	3	1

Content validity was assessed using cognitive interviewing and the open-ended question in the feedback form (*n* = 51). Four questions needed to be further clarified. The first question asking if the healthcare team included a nurse practitioner was confusing because some participants were not sure what a nurse practitioner role was. The second and third questions asked about overlapping roles in the healthcare team and the role of patients in the healthcare team. Respondents believed these questions needed to be expanded. The fourth question asked about annual household income. Respondents believed that rather than including a range for family income, it would be easier to respond to the question if it centered on how patients and families perceived their financial situation.

Overall, patients and families rated Team Processes 5.4/7 ± 0.9 (range: 1.6–7) with Boundary Work rated highest (mean: 5.6/7 ± 1.5) followed by PTE-Overall (mean: 5.4/7 ± 0.9), and Role Clarity (mean: 5/7 ± 1.3). Outcomes were rated on average 5.4/7 ± 1.1. The ratings for the dimensions included in the Team Processes ranged from 4.7/7 ± 1.9 for care coordination to 6.1/7 ± 1.2 for the BE score. The rho coefficients (r_s_) for the dimensions included in Team Processes ranged from 0.55 to 0.79 (*p* value< 0.001) (See Table 4). The mean values of Team Processes were generally lower for the primary care-pediatrics group with the exception of decision-making and care coordination which were lower in the maternal-perinatal care group (data not shown).

Construct validity was assessed using known groups (See Table 7). As anticipated, no differences were noted between men and women; patients and families; English and French language respondents; according to marital status; and employment status. As hypothesized, differences were identified in groups according to education, length of follow-up, and reason of consultation. We had anticipated differences according to team size and in teams with and without NPs but no significant differences were identified in these groups. Completion rates were similar for respondents who had and had not completed high school education. We were unable to assess differences for urban and non-urban respondents due to the small sample size.

**Table 7:** Differences in Team Processes and Outcomes by Known Group Comparisons^a,b^ in Study 1

	Specialty			Education	Follow up	Reason of Consultation	
Subscale Factor	Primary Care-Peds	Maternal Care	Oncology	Completed Graduate/Professional Degree	≥60 months	Pregnancy	New Health Problem
Boundary Work Trust	0.005	0.006	< 0.001		0.006		
Team Processes	0.003	0.001	< 0.001	< 0.001		0.002	
Role Clarity	0.008		0.003		0.011		
PTE-Overall	0.001	0.001	< 0.001	< 0.001	0.016	0.002	0.006
Outcomes	0.01	0.016	< 0.001				

^a^Mann Whitney U post hoc test

^b^Bonferroni correction significance α/n_groups_

*Responsiveness.* As anticipated, differences (*p* <  0.001) were noted for Boundary Work, Team Processes, Outcomes, and Role Clarity between low and high functioning teams (See Table 8).

**Table 8:** Differences in Processes and Outcomes in Low and High Functioning Teams in Study 1

					Mann-Whitney-U test	
Subscale Factor	Belief about Team Effectiveness	n	Mean	Standard Deviation	Median	*p* value
Boundary Work Trust	Low (Scores 1–5)	34	4.4	1.47	4	< 0.001
	High (Scores 6–7)	282	5.8	1.41	6	< 0.001
Team Processes	Low (Scores 1–5)	34	4	1.1	3.9	< 0.001
	High (Scores 6–7)	282	5.6	0.73	5.7	< 0.001
Outcomes	Low (Scores 1–5)	34	4.2	1.27	4.3	< 0.001
	High (Scores 6–7)	282	5.6	0.89	5.8	< 0.001
Role Clarity	Low (Scores 1–5)	34	3.8	1.03	4	< 0.001
	High (Scores 6–7)	282	5.1	1.22	5	< 0.001

### Changes made following study 1

Study 1 allowed us to refine the items included in the questionnaire and identify unclear or unnecessary questions. Using cognitive interviewing and respondent suggestions, changes were made to simplify and improve the flow of the questionnaire. The No opinion/Not applicable response option was not identified as an issue by respondents during the cognitive interviews, and the response was used infrequently by respondents who completed the questionnaire. We opted to remove the option to simplify the questionnaire, thus reducing the number of response options to six instead of seven. Other modifications included adding items to measure care coordination and an item pertaining to the team’s in-depth knowledge and skill, and separating the question describing patient and family’s role in the team into two questions to take into account that patients and families believed they had a role in the team and that they could play an active role as a team member. Respondents believed there were too many negatively-worded questions. We revised and left only three negatively worded items. We changed the monetary values for family income to asking about the perceived adequacy of family income, and added a definition for the NP role to ensure that participants understood which provider role was under consideration. The revised items are included in Table 9. The complete instrument included 43 items and is provided as an additional file.

**Table 9:** Revised Items Related to Healthcare Team Processes and Outcomes in Study 2

Items	
Role Clarity	The roles of members of the healthcare team are well-defined
	I am happy with the way work is divided among members of the healthcare team
Boundary Work	I trust all the members of the healthcare team
Perceptions of Team Effectiveness	
	My healthcare team is effective to provide healthcare
Decision-making	Team members share relevant information to help me make decisions about my healthcare
Decision-making	My ideas, information or observations are valued by members of my healthcare team
Communication	The plan of care and care objectives are clearly outlined in my chart
Communication	The test results and consultations are updated in my chart
Communication	The flow of information between team members, patients, friends and families is constrained
Coordination	I am aware of the next steps in my plan of care
Coordination	My healthcare team adjusts treatments according to changes in my condition
Coordination	My healthcare is well-organized
Cohesion	Team members work together to solve my healthcare issues
Problem-solving	Differences of opinions among team members are respected
Patient-Family Focus	I have a role to play in the healthcare team
Patient-Family Focus	My contribution is valued by members of the healthcare team
Patient-Family Focus	Working with friends or families to solve patient care issues is not part of the team’s mandate
Outcomes	Patient care is delivered in a timely manner
	Potential or actual complications are dealt with quickly by the team
	I return home with unanswered questions about my medication
	All relevant information is available to my healthcare team if I need to consult another healthcare provider or if I am hospitalized on another unit
	I return home with all my questions answered about my care
	Members of the healthcare team possess in-depth knowledge and the skills required to provide care

### Additional evidence of validity

#### Results from study 2

We used the first 242 completed questionnaires from a larger study of patients and families followed by primary care teams with nurse practitioners. These respondent were randomly selected from lists of patients. Data collection for Study 2 began in April 2018 and is ongoing. Dillman’s tailored approach was used to mail-out questionnaires to respondents and included a pre-notification, complete survey package with a $5 token incentive and a pre-paid return envelope, one reminder post-card, and a final complete package with a postage-paid retun envelope [62]. Respondents included patients (*n* = 161) and families (*n* = 74). Seventy percent of respondents were women. Overall, Boundary Work was rated highest 5.3/6 ± 0.92. Team Processes averaged 4.9/6 ± 0.66 with Patient/Family Focus scoring lowest (4.3/6 ± 1.13) and beliefs about team effectiveness (BE) scoring highest (5.4/6 ± 0.86). Outcomes of team functioning scored on average 5.1/6 ± 0.74. The rho coefficients (r_s_) for the dimensions included in Team Processes ranged from 0.61 (problem-solving) to 0.83 (care coordination) (*p* value< 0.001). Cronbach α values increased for PTE-Overall (0.88) and Outcomes (0.78), and were unchanged for Team Processes (0.84). The differences between low and high functioning teams for Boundary Work, Team Processes, Outcomes, and Role Clarity noted in Study 1 remained significant (*p* <  0.001) (Table 10).

**Table 10:** Differences in Processes and Outcomes in Low and High Functioning Teams in Study 2

					Mann-Whitney-U test	
Subscale Factor	Belief about Team Effectiveness	n	Mean	Standard Deviation	Median	*p* value
Boundary Work Trust	Low (Scores 1–4)	13	3.2	1.68	3	< 0.001
	High (Scores 5–6)	218	5.5	0.631	6	< 0.001
Team Processes	Low (Scores 1–4)	13	3.6	0.73	3.9	< 0.001
	High (Scores 5–6)	218	5	0.55	5.1	< 0.001
Outcomes	Low (Scores 1–4)	13	3.7	0.86	3.5	< 0.001
	High (Scores 5–6)	218	5.2	0.65	5.2	< 0.001
Role Clarity	Low (Scores 1–4)	13	3.5	1.35	4	< 0.001
	High (Scores 5–6)	218	5.4	0.61	5.5	< 0.001

#### Results from study 3

Participants (*n* = 50) were recruited as part of a prospective observational study examining the impact of follow-up by physiotherapists in primary care on patients’ pain levels for different musculo-skeletal conditions. Data were collected between October 1st 2016 and June 10th 2017. Respondents were primarily women (*n* = 37, 71%). A majority of participants were married (*n* = 31, 60%) and 35% (*n* = 18) were of low socio-economic status. Patients received an average of three therapy sessions with the physiotherapists. Similar results were found in this group of patients where Boundary Work was rated highest (5.3/6 ± 0.80), followed by PTE-Overall (5.1/6 ± 0.67), and Outcomes (5.0/6 ± 0.68). Team Processes averaged 5.1/6 ± 0.67 with Patient/Family Focus scoring lowest (4.7/6 ± 0.81) and beliefs about team effectiveness (BE) scoring highest (5.4/6 ± 0.80). The rho coefficients (r_s_) for the dimensions included in Team Processes ranged from 0.64 (patient/family focus) to 0.86 (communication) (*p* value< 0.001). Cronbach α values in this group were higher for PTE-Overall (0.94) and Team Processes (0.92), and slightly lower for Outcomes (0.76).

## Discussion

The purpose of the study was to develop and assess the psychometric properties of English and French language versions of the Patient-PTE questionnaire. The questionnaire was designed to measure patient and family perceptions of how healthcare teams provide care and the teams function in acute and primary care. We identified strong positive correlations between processes in teams and care outcomes that were consistent with a conceptual framework. Reliability indices for the revised questionnaire were good to excellent. The psychometric evaluation of the questionnaires provided evidence of validity.

The changes made to the questionnaire improve our ability to assess team functioning and distinguish between low and high functioning teams. Researchers can use the questionnaire to assess patient and family perceptions of processes in teams for different provider groups (e.g., physiotherapists, respiratory therapist, social workers) by changing the definition of the provider group being considered. This will allow researchers, decision-makers, patients and their families to understand how these providers contribute specifically to team processes and effective team functioning. We added a “Prefer not to respond” option to the question examining gender. Although no respondent asked for the addition, we believe that this will show respect for participants who do not wish to identify their gender or who do not identify with a specific gender. This will allow us to further refine our analyses and better understand the impact of gender on perceptions of team functioning [94]. In addition, we bolded key terms in the negatively worded items (e.g., not) in the Team Processes subscale to ensure that respondents clearly identify the negative statements.

The questionnaire helps to fill an important gap in the literature related PREM and PROM measures as well as the assessment of team functioning by placing patients and families at the center of the healthcare team conceptually and in the measurement tool [46, 95]. The questionnaire provides individual patient- and family-level measures of team processes and outcomes, and can help identify specific areas of team functioning that can be improved. The questionnaire can provide managers and decision makers with before and after data following the implementation of an intervention to improve team functioning or measure patient participation in increasingly complex healthcare teams [96].

The development of high functioning teams is a priority worldwide [27, 97, 98]. An important consideration to assess processes at the team-level is the ability to aggregate individual patient data to the team or organizational level and reduce the risk of measurement bias in aggregate data [88, 99]. To do this, it is necessary to determine the level of agreement of the assessments among the individuals within the same group [100]. Given the tool’s performance, it is possible to aggregate the individual level data to the team-, organizational- or system- level. The aggregated team or organizational assessments of team functioning can inform healthcare decisions (e.g., communication). This information about team processes has the potential to fill a gap in knowledge where the link between structures and outcomes is not always clear.

The questionnaire is short and easy to complete which is an important consideration for patients who are very ill, and families with limited time or energy to complete these instruments. It provides evidence that is consistent with the propositions in the conceptual framework about structures, team processes and outcomes. The questionnaire has a broad application because it measures patient and family views of team functioning in acute and primary care teams and as patients transition through the healthcare system. Patients and families can use the information to assess how their healthcare team is performing and determine the role they are playing in the team. Providers can use the information to tailor care, and support patient involvement as team members. Decision-makers can use aggregated data to monitor team performance. This appears to be particularly important in the current global trend of organizational restructuring and increased health system integration where processes in teams may be affected as teams work across settings, and healthcare systems around the world are becoming more goal-oriented and performance-driven [101, 102].

Some limitations must be kept in mind. Few responded to the questionnaire on-line. Thus, we were unable to examine if their perceptions differed from those who completed paper copies. This may be due in part to the electronic link to the questionnaire in Study 1 that was long to memorize, and the application for smart phones was not available. Future studies will need to recruit patients and families with different characteristics (e.g., hospitalized patients, patients in rural areas) as well as examine PTE from the provider perspective. These studies will allow us to gather additional evidence of validity for the revised questionnaire and test relationships between different variables in the framework and in low and high functioning teams. To test the relationship between perceptions of outcomes and actual outcomes, further research is needed to examine patient and family perceptions of outcomes and clinical or condition-specific indicators for health conditions including glycated hemoglobin A1C for patients with diabetes, systolic and diastolic blood pressure measures for patients with hypertension, adherence to medication regimens for patients with different health conditions.

## Conclusion

The study aimed to develop and assess the psychometric properties of the Patient-PTE questionnaire. The study produced evidence to support propositions in the conceptual framework of role enactment and perceptions of team effectiveness. The questionnaire can be used to assess structures, processes and perceived outcomes in low and high functioning teams in acute and primary care from the perspective of patients and their families. The instrument was validated for English and French language respondents. Individual patient-level data can be aggregated to the team-, organization- or system levels. Further research is needed to examine the perspectives of patients and families who are hospitalized or living in rural or remote areas and when other providers are included in the healthcare team.

## Abbreviations

∝: (Alpha)
AHRQ: Agency for Healthcare Research and Quality
IOM: Institute of Medicine
MS: Mean square
NPs: Nurse practitioners
PTE: Perceptions of team effectiveness
SD: Standard deviation
US: United States
